# Physiological bio-distribution of ^68^Ga-DOTA-TATE in pediatric patients

**DOI:** 10.1007/s12149-025-02040-9

**Published:** 2025-03-19

**Authors:** Nuh Filizoglu, Salih Ozguven, Selin Kesim, Kevser Oksuzoglu, Feyza Caglıyan, Tunc Ones, Fuat Dede, Halil Turgut Turoglu, Tanju Yusuf Erdil

**Affiliations:** 1 Department of Nuclear Medicine, University of Health Sciences, Kartal Dr. Lutfi Kirdar City Hospital, D-100 Güney Yanyol No:47 Cevizli Mevkii, Kartal, 34865 Istanbul, Turkey; 2 Department of Nuclear Medicine, Marmara University Pendik Training and Research Hospital, Istanbul, Turkey

**Keywords:** ^68^Ga-DOTA-TATE, PET/CT, Somatostatin receptors, Pediatric patients, Growth plates

## Abstract

**Objective:**

Somatostatin receptors (SSTRs) are G protein-coupled transmembrane receptors that serve as a specific molecular target for a number of radiopharmaceuticals utilized for the imaging of neuroendocrine tumors (NETs). ^68^Ga-DOTA-TATE is a somatostatin analog that demonstrates a high affinity for SSTR2. Pediatric malignancies, such as neuroblastoma, pheochromocytoma, and paraganglioma, have been shown to express SSTR2, and ^68^Ga-DOTA-TATE is currently being used to evaluate these pediatric neoplasms. We aimed to analyze the distribution pattern of ^68^Ga-DOTA-TATE based on age and location in pediatric patients.

**Methods:**

We retrospectively analyzed 247 consecutive ^68^Ga-DOTA-TATE whole-body PET/CT scans performed in our department from May 2015 to April 2024 in pediatric patients with known or suspected neuroblastoma, neuroendocrine malignancy, pheochromocytoma, and paraganglioma. 93 subjects were included in this study who were disease-free at the time of imaging and had no tracer-avid lesion on ^68^Ga-DOTA-TATE PET/CT. The patients were divided into four groups according to age: infant (0–2 years), pre-school (3–6 years), school (7–12 years), and adolescent (13–18 years). A comparison of the SUV values of each organ across age groups was performed.

**Results:**

The highest levels of physiological uptake were observed in the spleen across all age groups, except for infants, who demonstrated the highest SUV values in the kidneys. ^68^Ga-DOTA-TATE uptake in the parotid glands, submandibular glands, thyroid gland, thymus, liver, spleen, adrenal glands, stomach, intestines, uterus, prostate, and testes demonstrated a statistically significant increase in the adolescent age group. In contrast to all internal organs, the lowest SUV max values were observed for all growth plates within the adolescent age group.

**Conclusion:**

This study presents the bio-distribution pattern of ^68^Ga-DOTA-TATE in pediatric patients, according to age and location. The ranges of the SUVmax and SUVmean values of ^68^Ga-DOTA-TATE obtained in the various organs are of paramount importance for accurately diagnosing malignancy in ^68^Ga-DOTA-TATE PET/CT studies.

**Supplementary Information:**

The online version contains supplementary material available at 10.1007/s12149-025-02040-9.

## Introduction

Somatostatin receptors (SSTRs) are G protein-coupled membrane receptors that are expressed on the cell surface of neuroendocrine cells. There are five distinct receptor subtypes that have been identified in humans [1]. SSTRs also represent a specific molecular target for several radiotracers that are used for the imaging of neuroendocrine tumors (NETs). Recently, the advent of new positron emission tomography (PET) tracers has enabled the possibility of PET/computed tomography (CT) imaging of SSTRs [2].

The somatostatin analogs Gallium-68 (^68^Ga)-DOTA-TOC (DOTA-Tyr^3^-octreotide), ^68^Ga-DOTA-NOC (DOTA-Nal^3^-octreotide), and ^68^Ga-DOTA-TATE (DOTA-Tyr^3^-octreotate) demonstrate varying degrees of affinity for SSTRs [3]. ^68^Ga-DOTA-TATE is a somatostatin analog that demonstrates high affinity for SSTR2, which is the most prevalent subtype observed in NETs [4]. Pediatric malignancies, such as neuroblastoma, pheochromocytoma, and paraganglioma, are known to express SSTR2 and ^68^Ga-DOTA-TATE is currently being used to evaluate these pediatric neoplasms [5]. However, SSTRs are not exclusive to NETs; they are also found in a wide range of normal tissues and organs, including the pituitary gland, adrenal glands, and kidneys [6]. These physiological sites exhibit varying degrees of ^68^Ga-DOTA-TATE uptake, which should not be mistaken for pathological sites. Hence, a comprehensive understanding of the bio-distribution of ^68^Ga-DOTA-TATE is imperative for the accurate interpretation of PET/CT imaging.

The number of studies delineating the role of ^68^Ga-DOTA-TATE PET/CT in the staging and management of pediatric malignancies has recently increased [7–9]. However, there is a lack of literature defining the physiological uptake patterns of ^68^Ga-DOTA-TATE in pediatric population [10].

The objective of this study is to investigate the normal distribution pattern and physiological variants of ^68^Ga-DOTA-TATE in pediatric patients on PET/CT imaging. This study provides information on the range of normal standard uptake values (SUV) of ^68^Ga-DOTA-TATE in different organs based on age and location in pediatric patients, which has not been previously documented in the literature.

## Materials and methods

### Study subjects

We retrospectively analyzed 247 consecutive ^68^Ga-DOTA-TATE whole-body PET/CT scans performed in our department from May 2015 to April 2024 in pediatric patients with known or suspected neuroblastoma, neuroendocrine malignancy, pheochromocytoma, and paraganglioma. 93 subjects who were disease-free at the time of imaging and had no tracer-avid lesion on ^68^Ga-DOTA-TATE PET/CT were enrolled in this study. Patients with a history of treatment with somatostatin and chemotherapy were excluded from the study.

This study was conducted with the approval of the institutional ethics committee (date: May 2024, no: 17.05.2024.631). Prior to undergoing the examination, each subject participating in the study provided written informed consent.

### Preparation of ^68^Ga-DOTA-TATE

^68^Ga-DOTA-TATE was synthesized via a completely automated process, employing a standardized labeling sequence. Initially, the germanium-68 (^68^Ge)/^68^Ga generator (iThemba Labs, SA) was eluted with 0.6 M hydrochloric acid. The obtained ^68^Ga fraction was then subjected to purification using a PS-H + cartridge, with the objective of concentrating and purifying ^68^Ga from any residual ^68^Ge. Subsequently, the purified ^68^Ga was eluted with 1.7 mL of 5 M sodium chloride into the reaction vial. Following this, 25 µg of DOTA-TATE (ABX, Germany) was dissolved in 3 mL of 1.5 M HEPES buffer solution in the reaction vial. The reaction was conducted at 100 °C for eight minutes. The C-18 light cartridge was then employed for the purification of the ^68^Ga-DOTA-TATE, with the resulting purified product being eluted with a solution of 1 mL ethanol and 1 mL water and transferred into a sterile vial. It is noteworthy that the radiochemical purity exceeded 95% in all instances, as determined by high-performance liquid chromatography.

### ^68^Ga-DOTA-TATE ımaging

All ^68^Ga-DOTA-TATE PET/CT procedures were conducted using GE Healthcare Discovery-16 LS PET/CT scanner. In the beginning of the study, a uniform dose of averaging 2 MBq/kg was administered intravenously due to the lack of specific pediatric guidelines for ^68^Ga-DOTA-TATE PET/CT [11]. Subsequently, the doses were adjusted in accordance with the updated EANM pediatric dosing card for ^68^Ga-DOTA-TATE. In accordance with the guideline, the calculation of doses was performed using the following formula: “A[MBq]Administered = BaselineActivity x Multiple”. Additionally, all administered doses adhered strictly to the minimum and maximum dose limits recommended by the guidelines [12]. Whole-body images were obtained 60 ± 10 min after injection. A CT scan was executed with a 16-slice multidetector scanner (specifications: 80 milliampere (mA), 140 kilovolt (kV), table speed of 27 mm/rotation, and slice thickness of 5.0 mm) from the top of the head through the feet. Subsequently, a standard three-dimensional whole-body PET scan was completed with a three-minute capture time per bed position (three to seven bed positions), encompassing the same region as the CT scan. The PET images were then reconstructed with and without attenuation correction employing the iterative algorithm. The resulting images were uploaded to a workstation (Advantage Windows Workstation 4.6; GE Advantage) for post-processing and further analysis.

### Image analysis

**Fig. 1:**
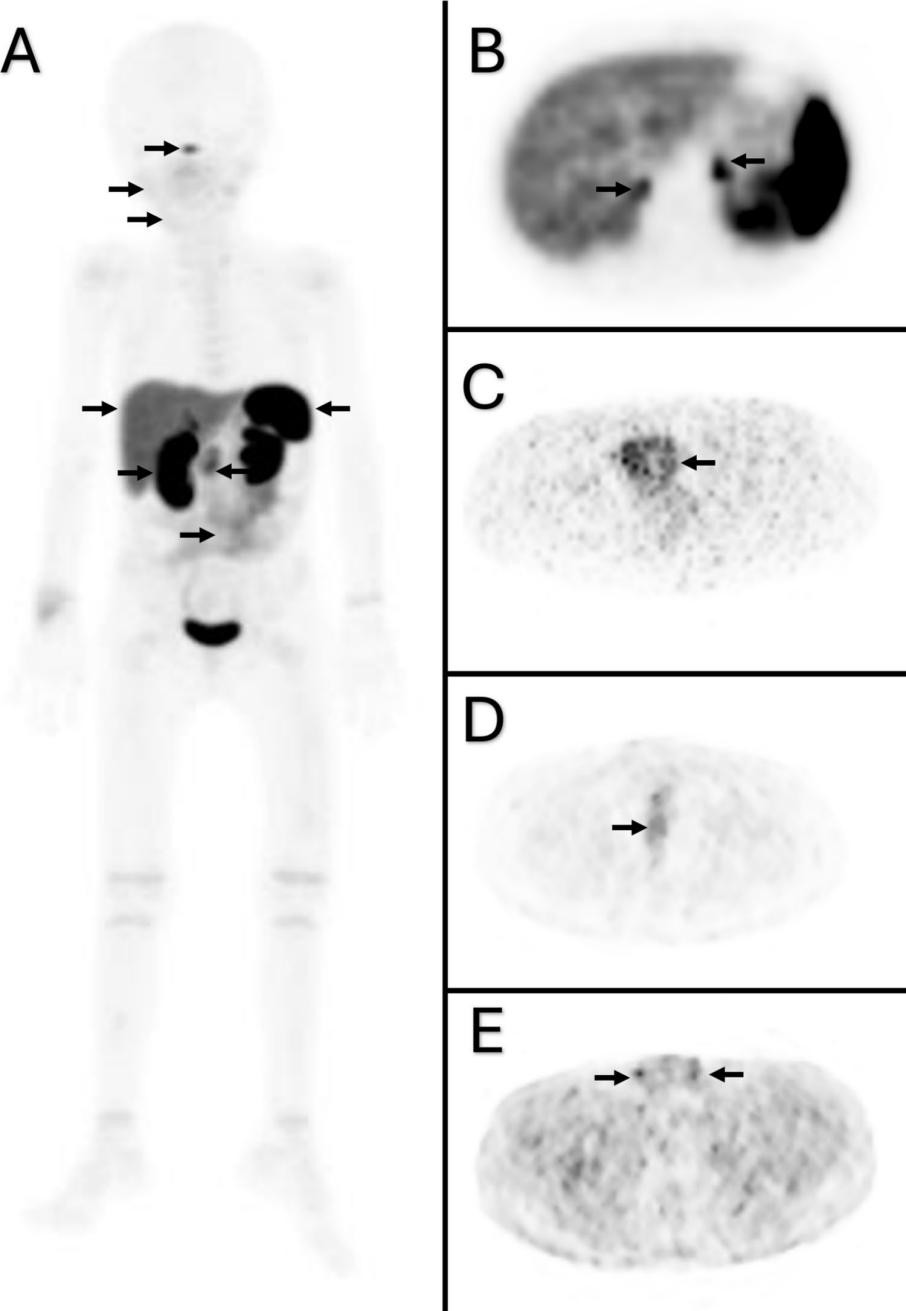
Physiological ^68^Ga-DOTA-TATE uptake in the pituitary gland, parotid gland, submandibular gland, spleen, pancreas, kidneys, and intestines is indicated by arrows in (**A**). Additionally, physiological ^68^Ga-DOTA-TATE uptakes in the adrenal glands (**B**), uterus (**C**), prostate (**D**), and testes (**E**) are also highlighted by arrows

Two nuclear medicine physicians evaluated ^68^Ga-DOTA-TATE PET/CT images. Maximum SUV (SUVmax) and mean SUV (SUVmean) values were calculated from a region of interest (ROI). Both two-dimensional (2D) and three-dimensional (3D) ROI delineation approaches were utilized based on the size and the anatomical characteristics of the organ or structure of interest. For smaller organs and structures (e.g., the skin and subcutaneous tissue), ROIs were defined as 2D regions on single axial CT slices.This approach ensured precise delineation of these thin structures while avoiding spillover effects from surrounding tissues. For larger organs, such as liver, spleen, and kidneys, three-dimensional volumes of interest (VOIs) incorporating multiple CT slices were employed. To ensure consistency, these 3D VOIs were designed to have a diameter of approximately 2 cm, thereby ensuring that the measurements captured a representative portion of the organ while maintaining anatomical accuracy. SUVmax was calculated as the maximum SUV value within the delineated ROI. SUVmean was calculated as the mean SUV concentration within the ROI. Lung ROI placement was conducted in the lower lobes, avoiding regions adjacent to the hilar vasculature. Kidney ROI placement was conducted in regions free of pelvicalyceal urinary activity. SUVmax and SUVmean values for a number of organs were calculated, including the pituitary gland, parotid glands, submandibular glands, thymus, thyroid gland, myocardium, lungs, breast, stomach, small intestine, colon, liver, spleen, pancreas head, pancreas body, right adrenal gland, left adrenal gland, right kidney, left kidney, prostate, uterus, testes, trapezius muscle, gluteal muscles, iliac crest, femora, subcutaneous tissue, skin, and growth plates (Fig. 1). The maximum SUVmax and SUVmean values are deemed to be the representative values for that organ.

### Statistical analysis

**Fig. 2:**
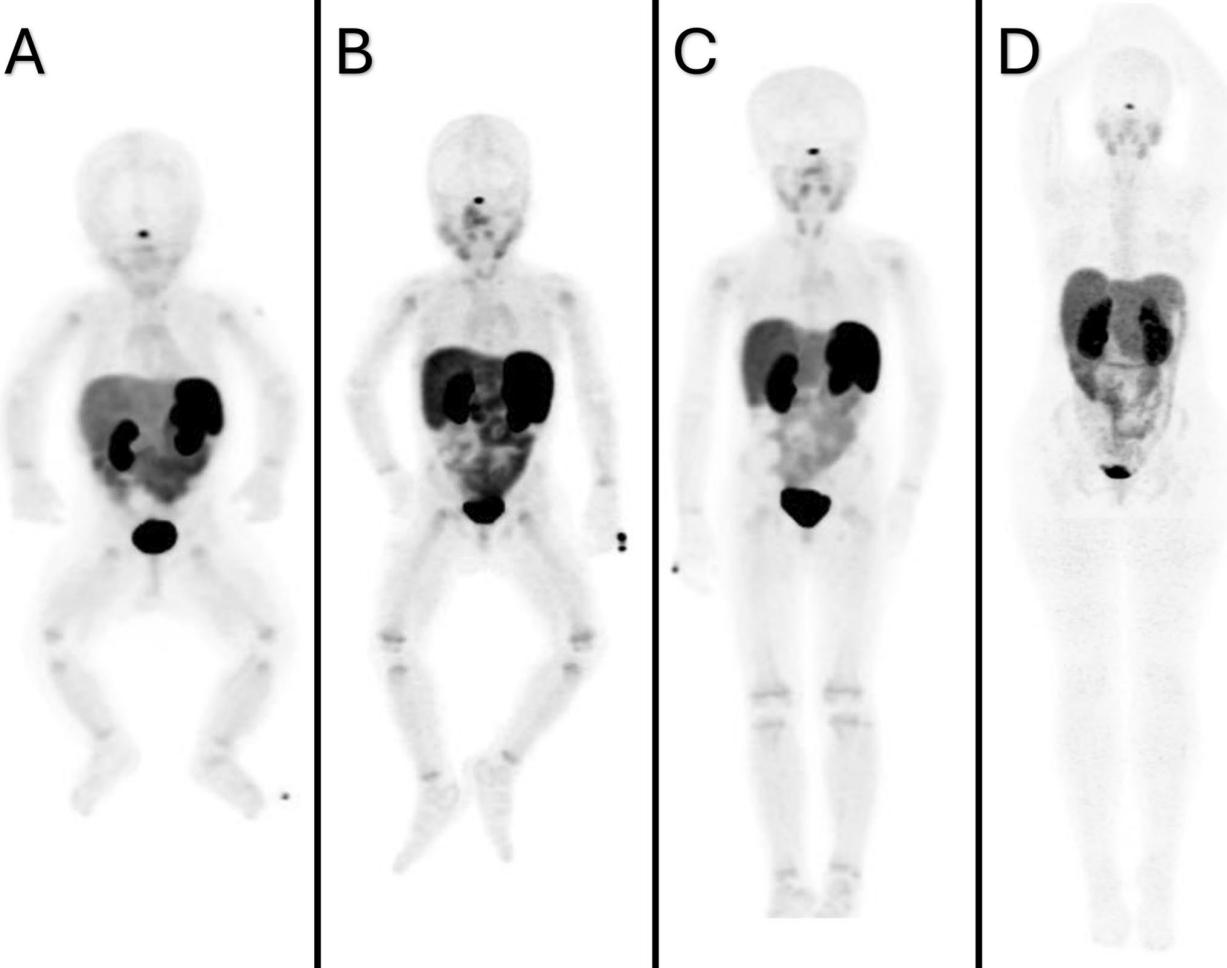
Maximum intensity projection (MIP) images representing each age group. **A** MIP image of 1-year-old boy, **B** MIP image of 4-year-old boy, **C** MIP image of 9-year-old girl, and **D** MIP image of 17-year-old girl

All data were analyzed with Statistical Package for Social Sciences (SPSS) software (version 25.0, SPSS Inc., Chicago/IL, USA). Referring to the previous studies [13], the patients were divided into four groups according to age: infant, 0–2 years (median age 1.0 yr, *n* = 28); pre-school, 3–6 years (median age 4.0 yr, *n* = 28); school, 7–12 years (median age 9.0 yr, *n* = 17); adolescent, 13–18 years (median age 16.0 yr, *n* = 20) (Fig. 2). Univariate descriptive statistics [mean, median, standard deviation (SD)] were calculated for each site and each age group. A comparison of the SUVmax values of each organ across age groups was conducted. As the number of patients in each group is less than thirty, the Shapiro–Wilk normality test was employed to ascertain whether the distribution of patients between groups was normal. A one-way analysis of variance (ANOVA) test was employed for parametric data and the Kruskal–Wallis test was utilized for nonparametric data to perform multiple comparisons of SUVmax at each site and in each age group. A p value of less than 0.05 was considered to be statistically significant.

## Results

From our cohort of 93 subjects, 48 patients (51.6%) were boy, and 45 patients (48.4%) were girl. The average age of the patients was 6.56 years (range 0–18 years; SD 5.54 years). Number of subjects by sex, mean age and mean weight values for the age groups are detailed in Table 1. The SUVmax values were categorized as high, moderate, mild, and minimal in accordance with the study of Moradi et al. [14]. The highest levels of physiological uptake were observed in the spleen across all age groups, with the exception of infants, which demonstrated the highest SUV values in the kidneys. High physiological uptake was also noted in the adrenal glands, pituitary gland, and liver. Moderate uptake was observed in the stomach, small intestine, pancreas head and body. Mild uptake (from minimal uptake to moderate uptake) was revealed in the salivary glands, thyroid gland, thymus, colon, internal genitals, and growth plates. Minimal uptake (average SUVmax < 1 g/mL) of tracer was observed in the gluteal and trapezius muscles, femora, iliac crest, breast tissue, lungs, myocardium, skin, and subcutaneous tissue. The average SUVmax (± SD) and the average SUVmean (± SD) for all the organs considered in each age group on ^68^Ga-DOTA-TATE PET/CT are summarized in Table 2.

**Table 1:** Baseline characteristics of age groups

Age group	Sex (M/F)	Mean age (years) ± SD	Mean weight (kg) ± SD
Infant	7/21	1.11 ± 0.83	9.92 ± 1.90
Pre-school	15/13	4.18 ± 1.15	17.96 ± 3.62
School	12/5	8.94 ± 1.71	30.82 ± 11.82
Adolescent	14/6	15.5 ± 1.53	63.45 ± 18.20

**Table 2:** Average SUVmax (± SD) and average SUVmean (± SD) values of ^68^Ga-DOTA-TATE in different age groups

Organ	Infant		Pre-school		School		Adolescent	
	SUVmax (± SD)	SUVmean (± SD)	SUVmax (± SD)	SUVmean (± SD)	SUVmax (± SD)	SUVmean (± SD)	SUVmax (± SD)	SUVmean (± SD)
Pituitary gland	6,19 (0,74)	3,74 (0,44)	7,14 (0,62)	4,52 (0,41)	8,92 (1,42)	5,87 (0,88)	10,1 (1,83)	6,57 (1,21)
Parotid gland	1,49 (0,13)	0,93 (0,07)	1,40 (0,11)	0,87 (0,06)	1,86 (0,24)	1,16 (0,15)	2,23 (0,2)	1,37 (0,13)
Submandibular	1,68 (0,15)	1,01 (0,09)	1,77 (0,15)	1,11 (0,1)	2,28 (0,34)	1,40 (0,22)	2,61 (0,25)	1,62 (0,26)
Thyroid gland	1,82 (0,16)	1,06 (0,09)	1,82 (0,13)	1,04 (0,07)	2,78 (0,46)	1,64 (0,29)	3,4 (0,36)	1,97 (0,22)
Thymus	1,35 (0,11)	0,84 (0,07)	1,26 (0,09)	0,76 (0,04)	1,51 (0,17)	0,91 (0,09)	2,06 (0,21)	1,16 (0,11)
Breast	0,48 (0,02)	0,30 (0,01)	0,45 (0,02)	0,28 (0,01)	0,48 (0,04)	0,27 (0,02)	0,58 (0,07)	0,34 (0,04)
Lungs	0,51 (0,03)	0,31 (0,02)	0,42 (0,02)	0,27 (0,02)	0,50 (0,03)	0,30 (0,02)	0,48 (0,04)	0,28 (0,02)
Myocardium	0,78 (0,04)	0,51 (0,03)	0,69 (0,04)	0,44 (0,02)	1,02 (0,15)	0,63 (0,08)	1,29 (0,14)	0,74 (0,07)
Stomach	2,85 (0,28)	1,85 (0,19)	3,08 (0,24)	1,95 (0,169	4,17 (0,51)	2,73 (0,35)	5,33 (0,57)	3,45 (0,37)
Liver	3,79 (0,29)	2,88 (0,22)	4,92 (0,25)	3,83 (0,21)	6,66 (0,52)	4,82 (0,37)	7,46 (0,56)	5,18 (0,40)
Spleen	14,85 (1,26)	10,70 (0,96)	18,02 (1,06)	13,56 (0,84)	22,88 (1,54)	15,82 (0,98)	25,39 (2,10)	17,70 (1,62)
Pancreas head	5,81 (0,78)	3,61 (0,47)	5,24 (0,32)	3,26 (0,22)	6,05 (0,55)	3,74 (0,32)	5,12 (0,49)	3,19 (0,30)
Pancreas body	3,76 (0,26)	2,66 (0,18)	3,78 (0,19)	2,54 (0,14)	4,16 (0,32)	2,76 (0,22)	3,53 (0,32)	2,25 (0,22)
Right adrenal	4,50 (0,46)	3,15 (0,32)	5,31 (0,32)	3,70 (0,24)	7,3 (0,56)	5,19 (0,40)	9,65 (0,85)	6,25 (0,50)
Left adrenal	5,45 (0,61)	3,88 (0,40)	5,33 (0,41)	3,65 (0,33)	7,71 (0,86)	5,09 (0,54)	9,67 (0,87)	6,23 (0,56)
Right kidney	21,17 (2,06)	12,33 (1,34)	16,36 (1,40)	9,56 (0,77)	15,86 (1,26)	9,09 (0,76)	15,84 (1,87)	8,94 (0,97)
Left kidney	26,59 (5,14)	15,29 (3,23)	16,21 (1,26)	9,34 (0,65)	15,95 (1,46)	9,60 (0,81)	16,46 (2,57)	9,77 (1,44)
Small intestine	4,01 (0,28)	2,84 (0,20)	3,45 (0,19)	2,39 (0,13)	4,33 (0,35)	2,79 (0,24)	4,91 (0,43)	3,14 (0,30)
Colon	1,50 (0,11)	0,94 (0,07)	1,15 (0,10)	0,71 (0,06)	1,19 (0,16)	0,70 (0,09)	1,84 (0,18)	1,09 (0,10)
Uterus	1,42 (0,12)	1,01 (0,08)	1,39 (0,12)	0,93 (0,08)	2,12 (0,31)	1,41 (0,19)	2,40 (0,47)	1,38 (0,23)
Prostate	1,35 (0,19)	0,84 (0,12)	1,28 (0,10)	0,87 (0,07)	1,76 (0,13)	1,07 (0,07)	3,68 (0,38)	2,21 (0,21)
Testes	0,64 (0,08)	0,39 (0,04)	0,65 (0,06)	0,41 (0,04)	0,66 (0,06)	0,39 (0,03)	1,50 (0,20)	0,83 (0,11)
Trapezius	0,65 (0,03)	0,45 (0,02)	0,56 (0,03)	0,36 (0,02)	0,55 (0,04)	0,34 (0,02)	0,73 (0,06)	0,43 (0,03)
Gluteus	0,67 (0,05)	0,44 (0,02)	0,53 (0,02)	0,36 (0,02)	0,67 (0,06)	0,39 (0,03)	0,82 (0,07)	0,49 (0,04)
Iliac crest	0,96 (0,05)	0,61 (0,03)	1,02 (0,10)	0,67 (0,06)	0,97 (0,06)	0,59 (0,03)	1,14 (0,09)	0,69 (0,04)
Femora	0,83 (0,04)	0,53 (0,03)	0,87 (0,05)	0,56 (0,03)	0,80 (0,05)	0,48 (0,03)	0,93 (0,08)	0,52 (0,03)
Subcutaneous	0,32 (0,02)	0,19 (0,01)	0,30 (0,02)	0,18 (0,01)	0,28 (0,02)	0,16 (0,01)	0,40 (0,03)	0,23 (0,01)
Skin	0,17 (0,01)	0,11 (0,01)	0,16 (0,01)	0,10 (0,01)	0,15 (0,02)	0,10 (0,01)	0,21 (0,02)	0,13 (0,01)

*SUVmax* maximum standard uptake values, *SUVmean* mean standard uptake values, *SD* standard deviation

The SUVmax values of each anatomical structure, with the exception of those exhibiting minimal uptake, were compared between each age group. ^68^Ga-DOTA-TATE uptake in the parotid glands, submandibular glands, thyroid gland, and thymus displayed a statistically significant increase in the adolescent age group (*p* = 0.036, *p* = 0.008, *p* = 0.002, *p* = 0.009, respectively). The highest SUVmax values for abdominal organs, including the liver, spleen, adrenal glands, stomach, and intestines, were also observed in the adolescent age group (*p* < 0.001, *p* < 0.001, *p* < 0.001, *p* < 0.001, *p* = 0.01, respectively). While SUVmax values for pancreas and kidneys did not show a statistically significant difference between age groups. The highest SUVmax values were recorded for the genital organs, including the uterus, prostate, and testes, in the adolescent age group (*p* < 0.005, *p* < 0.001, *p* < 0.001, respectively). Table 3. provides a summary of the change in SUVmax values for all organs examined in each age group.

**Table 3:** Comparison of average SUVmax values of each organ according to age groups

Organ	Infant		Pre-school		School		Adolescent		*p* value
	Mean (± SD)	Median	Mean (± SD)	Median	Mean (± SD)	Median	Mean (± SD)	Median	
Pituitary gland	6,19 (0,74)	4,56	7,14 (0,62)	7,19	8,92 (1,42)	7,09	10,1 (1,83)	7,15	0,185
Parotid gland	1,49 (0,13)	1,35	1,40 (0,11)	1,26	1,86 (0,24)	1,64	2,23 (0,2)	2,28	**0,008**
Submandibular	1,68 (0,15)	1,54	1,77 (0,15)	1,66	2,28 (0,34)	2,17	2,61 (0,25)	2,99	**0,036**
Thyroid gland	1,82 (0,16)	1,53	1,82 (0,13)	1,73	2,78 (0,46)	2,03	3,4 (0,36)	3,31	**0,002**
Thymus	1,35 (0,11)	1,20	1,26 (0,09)	1,09	1,51 (0,17)	1,46	2,06 (0,21)	1,77	**0,009**
Stomach	2,85 (0,28)	2,48	3,08 (0,24)	2,81	4,17 (0,51)	3,70	5,33 (0,57)	4,83	** < 0,001**
Liver	3,79 (0,29)	3,4	4,92 (0,25)	4,85	6,66 (0,52)	6,20	7,46 (0,56)	7,04	** < 0,001**
Spleen	14,85 (1,26)	13,71	18,02 (1,06)	17,06	22,88 (1,54)	22,32	25,39 (2,10)	25,45	** < 0,001**
Pancreas head	5,81 (0,78)	4,68	5,24 (0,32)	4,79	6,05 (0,55)	5,99	5,12 (0,49)	4,72	0,460
Pancreas body	3,76 (0,26)	3,61	3,78 (0,19)	3,72	4,16 (0,32)	4,34	3,53 (0,32)	3,39	0,401
Right adrenal	4,50 (0,46)	4,33	5,31 (0,32)	5,05	7,3 (0,56)	7,78	9,65 (0,85)	9,12	** < 0,001**
Left adrenal	5,45 (0,61)	4,42	5,33 (0,41)	4,98	7,71 (0,86)	7,01	9,67 (0,87)	9,73	** < 0,001**
Right kidney	21,17 (2,06)	17,05	16,36 (1,40)	13,90	15,86 (1,26)	16,48	15,84 (1,87)	14,26	0,154
Left kidney	26,59 (5,14)	19,98	16,21 (1,26)	15,17	15,95 (1,46)	16,61	16,46 (2,57)	13,18	0,068
Small intestine	4,01 (0,28)	4,06	3,45 (0,19)	3,51	4,33 (0,35)	3,89	4,91 (0,43)	4,76	**0,010**
Uterus	1,42 (0,12)	1,39	1,39 (0,12)	1,45	2,12 (0,31)	2,35	2,40 (0,47)	2,09	**0,005**
Prostate	1,35 (0,19)	1,22	1,28 (0,10)	1,25	1,76 (0,13)	1,59	3,68 (0,38)	3,36	** < 0,001**
Testes	0,64 (0,08)	0,62	0,65 (0,06)	0,61	0,66 (0,06)	0,63	1,50 (0,20)	1,15	** < 0,001**

*SUVmax* maximum standard uptake values, *SD* standard deviation

*p* values less than 0.05 are presented in bold to indicate statistically significant differences among the age groups (infant, pre-school, school, and adolescent)

**Fig. 3:**
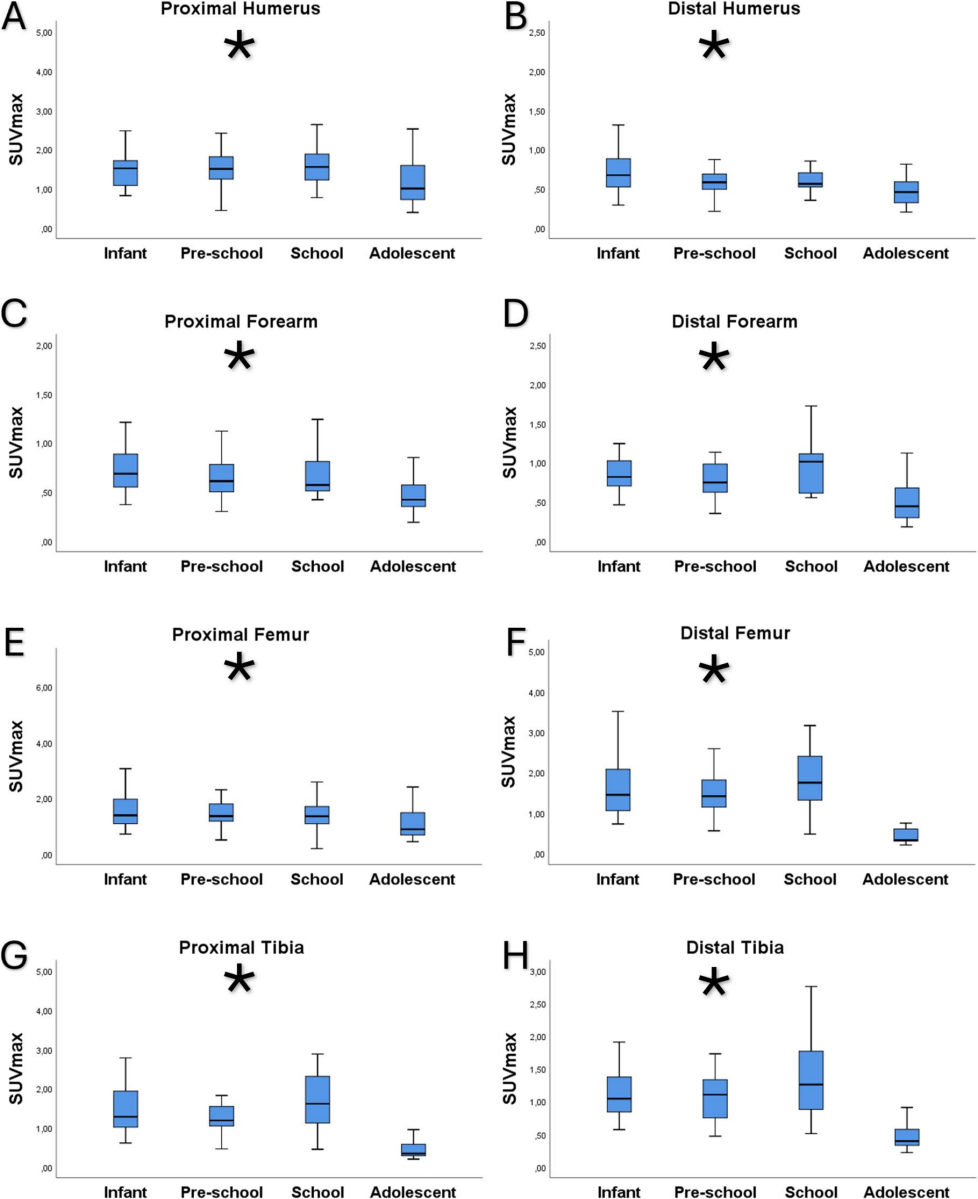
Box plots of each growth plate demonstrating the change of SUVmax according to age group. **A** proximal humerus, **B** distal humerus, **C** proximal forearm, **D** distal forearm, **E** proximal femur, **F** distal femur, **G** proximal tibia, and **H** distal tibia. Asterisks are used to denote statistically significant differences between all age groups (infant, pre-school, school, and adolescent) with *p* values less than 0.05

Upon examination of the growth plates in each age group, it was observed that the distal femur exhibited the highest SUVmax values in both the infant and school-age groups. In the pre-school age group, the proximal femur exhibited the highest SUVmax values. Lastly, proximal humerus demonstrated the highest SUVmax values in the adolescent age group. The SUVmax values of each growth plate, were also compared between each age group (Fig. 3). Contrary to all internal organs, the lowest SUV max values were recorded for all growth plates in the adolescent age group (Table 4).

**Table 4:** Comparison of average SUVmax values of each growth plate according to age groups

Age group	Infant		Pre-school		School		Adolescent		*p* value
Organ	Mean (± SD)	Median	Mean (± SD)	Median	Mean (± SD)	Median	mean (± SD)	Median	
Proximal humerus	1,51 (0,10)	1,51	1,61 (0,12)	1,50	1,65 (0,15)	1,55	1,15 (0,13)	0,89	**0,032**
Distal humerus	0,71 (0,05)	0,67	0,65 (0,07)	0,58	0,58 (0,03)	0,56	0,46 (0,03)	0,45	**0,006**
Proximal forearm	0,73 (0,04)	0,68	0,68 (0,05)	0,61	0,65 (0,05)	0,57	0,45 (0,04)	0,42	** < 0,001**
Distal forearm	0,93 (0,07)	0,81	0,83 (0,06)	0,74	1,00 (0,09)	1,01	0,51 (0,05)	0,44	** < 0,001**
Proximal femur	1,57 (0,11)	1,38	1,63 (0,18)	1,35	1,47 (0,16)	1,35	1,08 (0,11)	0,88	**0,028**
Distal femur	1,63 (0,13)	1,45	1,55 (0,13)	1,41	1,78 (0,18)	1,75	0,43 (0,05)	0,33	** < 0,001**
Proximal tibia	1,49 (0,13)	1,28	1,34 (0,13)	1,18	1,61 (0,18)	1,61	0,42 (0,04)	0,34	** < 0,001**
Distal tibia	1,16 (0,08)	1,04	1,10 (0,09)	1,10	1,38 (0,15)	1,26	0,47 (0,04)	0,39	** < 0,001**

*SUVmax* maximum standard uptake values, *SD* standard deviation

*p* values less than 0.05 are presented in bold to indicate statistically significant differences among the age groups (infant, pre-school, school, and adolescent)

## Discussion

To the best of our knowledge, this is the first study to evaluate the physiological bio-distribution of ^68^Ga-DOTA-TATE in pediatric patients. Furthermore, it elucidates the ranges of SUVmax and SUVmean values of ^68^Ga-DOTA-TATE in different organs, and their variations across different age groups. High physiological uptake of ^68^Ga-DOTA-TATE was documented in spleen, kidneys, adrenal glands, pituitary glands, and liver in all age groups. Moderate levels of uptake were identified within the stomach, small intestine, pancreas head, and pancreas body. Mild uptake was observed in salivary glands, thyroid gland, thymus, colon, internal genitalia, and growth plates. Lastly, minimal activity was observed in the gluteal and trapezius muscles, femora, iliac crest, breast tissue, lungs, myocardium, skin, and subcutaneous tissue.

In this study, the distribution of ^68^Ga-DOTA-TATE was analyzed from the vertex to the toes and the results demonstrated that the pituitary gland exhibited the highest uptake of ^68^Ga-DOTA-TATE within the head region, which can be explained by the presence of SSTR2 in the pituitary gland’s anterior lobe cells [15]. Furthermore, no significant differences in ^68^Ga-DOTA-TATE uptake were observed between age groups. Nevertheless, activity uptake was not observed in the cranium other than in the pituitary gland. Despite the presence of both SSTR1 and SSTR2 in the cerebral cortex, the limbic system, the paraventricular nuclei of the hypothalamus, and the basal ganglia, ^68^Ga-DOTA-TATE is unable to cross the blood–brain barrier [16]. Therefore, ^68^Ga-DOTA-TATE uptake was not observed in the brain. The salivary glands, including the parotid and submandibular glands, exhibited mild uptake of ^68^Ga-DOTA-TATE, which is consistent with the findings of Anzola et al. who demonstrated that SSTRs are abundantly present in the salivary glands [17]. Moreover, ^68^Ga-DOTA-TATE uptake in the salivary glands displayed a statistically significant increase in the adolescent age group (*p* = 0.036, *p* = 0.008). This can be attributed to the increasing weight and vascularity of the salivary glands with age [18].

In the neck region, the thyroid gland showed mild uptake of ^68^Ga-DOTA-TATE in our study, and the median ^68^Ga-DOTA-TATE uptake increased significantly with age (*p* = 0.002). The presence of SSTR2 in both normal and pathological thyroid tissue can be explanation for this observation. Thyroid adenomas, autoimmune thyroid diseases and multinodular goiters have been documented to increase the uptake of ^68^Ga-DOTA-TATE [19]. Understandably, higher SUVmax values are observed in adolescents since the increase in the number of thyrocytes with age [20].

In the thorax, minimal ^68^Ga-DOTA-TATE uptake was noted in the lung and myocardium. SSTR2 is found on several component of pulmonary inflammation including epithelial cells, inflammatory cells and possibly fibroblasts. In contrast, normal lung tissue has predominantly SSTR4, which binds to ^68^Ga-DOTA-TATE with less affinity, and in the absence of inflammation, lung tissue shows minimal uptake of ^68^Ga-DOTA-TATE [21]. Although molecular studies have demonstrated SSTR expression in normal myocardium, it has been reported that there is no significant physiological uptake of ^68^Ga-DOTA-TATE peptides in the myocardium, as in our study [22]. In addition, as breast glandular tissue does not express significant SSTR2, there was also no substantial uptake of ^68^Ga-DOTA-TATE in the breast [23]. Previous studies have demonstrated that somatostatin plays a distinct role in the development of the human immune system [24]. SSTR expression has been observed in a wide range of thymic epithelial cells. The maturation of thymic epithelial cells with age has been associated with an increase in SSTR expression in these cells [25]. Our findings align with those of previous studies, as the highest SUVmax values for the thymus were recorded in the adolescent age group (*p* = 0.009). Following the completion of puberty and the involution of the thymus, no evidence of SSTR expression was identified in the thymus. Consequently, adult patients display no evidence of thymic activity in ^68^Ga-DOTA-TATE PET/CT imaging [26].

The spleen is composed of two main components: the red pulp and the white pulp. It has been demonstrated that SSTRs are predominantly present in the red pulp of the spleen. Additionally, Reubi et al. demonstrated that the red pulp contains extensively distributed SSTRs. SSTR2 is the most prevalent SSTR subtype observed in the spleen [27]. Consequently, the spleen exhibited marked ^68^Ga-DOTA-TATE uptake, as anticipated, resulting in the highest SUV values for all age groups except infants. In addition, there was a statistically significant correlation between increasing age and a corresponding increase in the average SUVmax values of the spleen (*p* < 0001). The relatively low physiological splenic uptake of ^68^Ga-DOTA-TATE in infants and young children may be attributed to incomplete or immature expression of the SSTRs during early life [24].

In our study, moderate to high ^68^Ga-DOTA-TATE uptake was observed in the liver, and the average SUV values showed a statistically significant increase with age (*p* < 0.001). Adolescents exhibited the highest SUVmax values, while infants recorded the lowest. Recent studies have revealed that hepatocytes and hepatic stellate cells express SSTR1 and SSTR2 [28]. Furthermore, the liver, playing a crucial role in peptide metabolism, is responsible for the removal of ^68^Ga-DOTA-TATE from the bloodstream, leading to its accumulation in the liver [29]. Our findings are consistent with those of previous studies, demonstrating a positive correlation between age and ^68^Ga-DOTA-TATE uptake by the liver in adult subjects. It is hypothesized that an increase in the number of hepatocytes and hepatic stellate cells with age leads to increased uptake of ^68^Ga-DOTA-TATE by the liver [30].

The distribution of ^68^Ga-DOTA-TATE was found to vary across the pancreas. A higher physiological activity in the uncinate process has been documented in several prior studies, attributable to the presence of subtypes 2, 3, and 5 of SSTR on islet cells and the comparatively higher concentration of islet cells in this section of the pancreas [31]. In accordance with the findings of previous studies, our investigation revealed elevated SUV values in the uncinate process of the pancreas comparing pancreas body [32]. The average SUV values for both the uncinate process and pancreas body between age groups were found to be similar, with no statistically significant variation observed.

An intense uptake of ^68^Ga-DOTA-TATE was also recorded in the adrenal glands. Based on the evidence presented by Epelbaum et al., the relatively high uptake observed in the adrenal glands can be attributed to the presence of five subtypes of SSTRs, predominantly SSTR2, expressed in adrenal tissue [33]. Additionally, the ^68^Ga-DOTA-TATE uptake in both adrenal glands demonstrated a statistically significant correlation with age, with the highest SUV values observed in adolescents. This finding could be explained by the ongoing maturation and growth of the adrenal glands during adolescence. During the course of normal human sexual development, the adrenal gland undergoes adrenarche, a maturational process characterized by an increase in the secretion of androgens and estrogens [34]. Since somatostatin plays a critical role in adrenal gland development, this maturation process leads to increased expression of SSTRs [35].

Irregular and heterogenous ^68^Ga-DOTA-TATE uptake was observed in the stomach and intestine. Bowel motility and movement artifacts, as well as the expression of SSTR2 at different rates in the entire gastrointestinal tract, may be the underlying causes of this irregular and variable uptake [36]. The average SUV values of both the stomach and intestine were observed to increase with age. This can be attributed to the age-related increase in D cells [37].

When evaluating the genitourinary system, the highest activity was indeed found in the bladder, but this is a result of accumulation of radioactive urine rather than true receptor-ligand interaction. Thus, we did not evaluate bladder SUV values. Excluding the bladder, the kidney has the highest tracer activity in the urogenital system. Reubi et al. proved that the vasa recta of the kidney expresses SSTR2 at a very high concentration [38]. SSTR2 receptors have also been identified in the tubular cells of the renal cortex, although at a lower density. In addition, DOTA peptide is filtered by the glomeruli, but it is also partly reabsorbed in the proximal tubule, leading to enhanced activity in the kidney [39]. Consequently, all these factors could be the most relevant explanation for the high SUV values in the kidneys. Nevertheless, the average SUV values in the kidney did not demonstrate any statistically significant differences between the age groups.

Previous studies have identified SSTRs, specifically in the stromal component of prostate tissue [40]. The expression of SSTR2 in the endometrium throughout the various phases of the menstrual cycle has also been well-documented in the literature [41]. Nonetheless, minimal activity was observed in the entire genital organs until the onset of adolescence. It is evident that the development of the genital organs during puberty results in an increase in ^68^Ga-DOTA-TATE uptake, and mild activity uptake was observed in the prostate, uterus, and testis in the adolescent age group.

A minimal ^68^Ga-DOTA-TATE uptake was observed in skeletal muscle and bone due to the low-level expression of SSTRs in osteoblasts and myoblasts [42]. However, it is established that SSTRs have been identified on the growth plate [43]. Consequently, mild ^68^Ga-DOTA-TATE uptake was detected on the growth plates of long bones. Considering that neuroblastoma, one of the most prevalent pediatric malignancies, commonly metastasizes to the metaphysis of long bones, it is crucial to establish a normal range of SUV values to discern between malignant and physiological ^68^Ga-DOTA-TATE uptake. However, there have been no studies describing physiological uptake of ^68^Ga-DOTA-TATE in the growth plates before. In this cohort, the distal femur exhibited the highest SUVmax values in both the infant and school-age groups. In the pre-school age group, the proximal femur demonstrated the highest SUVmax values. Eventually, proximal humerus showed the highest SUVmax values in the adolescent age group. Otani et al. documented that distal femur has the highest SUV value among all growth plates on ^18^F-FDG PET/CT [44]. Nevertheless, this study was conducted using ^18^F-FDG PET/CT, and the age groups differed from those in our study. Lastly, the average SUV values of growth plates at all sites decreased with age, and the lowest average SUV values were documented in the adolescent age group. This result could be readily explained by the fact that the growth plate closes during puberty [45].

The present study reveals noteworthy disparities in the physiological bio-distribution of ^68^Ga-DOTA-TATE between pediatric and adult subjects. A previous study by Özgüven et al. examined the bio-distribution of ^68^Ga-DOTA-TATE in normal adult subjects, highlighting the physiological uptake patterns in various organs [46]. In contrast, our study in pediatric patients unveils distinct variations attributable to developmental and physiological differences between children and adults. One significant finding is that the uptake of ^68^Ga-DOTA-TATE in the spleen, liver, and adrenal glands of pediatric subjects is comparatively lower than that in adults. This discrepancy might be attributed to the differing levels of somatic and metabolic activity in these organs among different age groups, and the comparatively reduced liver uptake could be indicative of variations in somatostatin receptor expression or hepatic metabolism with age. A similar rationale underlies the reduced uptake observed in the adrenal glands of pediatric subjects, which is expected to be associated with the maturation process of the adrenal cortex, a process that differs between pediatric and adult populations. A notable distinction is the lower uptake observed in the genital organs of pediatric patients, which is expected given the underdevelopment of these organs before puberty. The absence of significant physiological activity in the genital organs of children likely contributes to the reduced tracer accumulation compared to adults. In contrast, our study revealed a relatively higher uptake of ^68^Ga-DOTA-TATE in the thymus of pediatric subjects, which aligns with the known fact that the thymus is more active and prominent during childhood, playing a crucial role in immune system development. However, as individuals age, the thymus undergoes involution, leading to a progressive decrease in both its size and metabolic activity, which can be attributed to the lower uptake observed in adult subjects. Furthermore, while the presence of growth plates was observed in pediatric patients, they were absent in adult subjects. This finding is of considerable physiological significance, as the presence of active growth plates in children is indicative of ongoing skeletal development. Conversely, the absence of growth plate uptake in adults is anticipated, given the completion of skeletal maturation. Moreover, our study identified a higher uptake in the kidneys of pediatric subjects compared to adults. This heightened uptake in the kidneys of pediatric subjects could potentially be ascribed to the elevated glomerular filtration rate and renal function observed in children, which have been demonstrated to influence tracer excretion and accumulation patterns. These differences highlight the necessity of considering age-related physiological variations when interpreting ^68^Ga-DOTA-TATE imaging results. The findings underscore the necessity for pediatric-specific reference data for accurate assessment and diagnosis of younger patients.

In this study, we observed that the uptake of certain organs, particularly those located near the bladder, may be overestimated due to spillover effects resulting from the high radioactivity within the bladder. This phenomenon is a well-documented limitation in PET/CT imaging, particularly in studies utilizing radiotracers like ^68^Ga-DOTA-TATE, which are excreted via the urinary system [47]. High bladder activity creates partial volume and spillover effects, where the detected activity in adjacent regions may reflect contamination from the bladder rather than true radiotracer uptake. Organs, such as prostate, uterus, and adjacent pelvic structures, are particularly susceptible to this effect due to their proximity to the bladder. The intensity of the spillover effect is influenced by factors, such as bladder filling, patient hydration status, and imaging acquisition timing. To mitigate this effect, several strategies have been proposed in the literature. One common approach is adequate pre-scan hydration and encouraging patients to void their bladder immediately before imaging [48]. In addition, some protocols also recommend administering a diuretic to accelerate urinary clearance and reduce bladder activity during imaging [49]. In our study, while patient preparation included hydration and bladder voiding before imaging, these measures may not have been sufficient to completely eliminate spillover effects. Overall, while the current findings are robust, the impact of bladder radioactivity on adjacent organ uptake should be acknowledged as a limitation. Furthermore, future research could focus on optimizing patient preparation protocols and exploring imaging corrections to minimize spillover effects and improve the reliability of quantitative PET/CT analysis.

A well-recognized limitation in nuclear medicine imaging is the partial volume effect, particularly affecting small organs whose size varies significantly during development.This effect arises due to the limited spatial resolution of imaging systems, which results in spill-out or spill-in of radiotracer signals, causing underestimation or overestimation of uptake values, respectively [50]. This effect poses a particular challenge in the evaluation of very small organs, such as those found in infants. In these cases, the degree of underestimation can be substantial, complicating the accurate assessment of physiological uptake changes associated with growth and development. For instance, significant volumetric changes may be observed in the thymus, adrenal glands, breast, and genital organs, which may result in variable radiotracer distribution patterns. The absence of adequate correction methods can lead to inaccuracies in the uptake measurements of these small structures, potentially resulting in misinterpretations of developmental trends [51]. A number of approaches have been advanced as a means of mitigating the impact of partial volume effect in small organ imaging. These include the development of recovery coefficient-based corrections, high-resolution imaging techniques, and partial volume correction algorithms, which have been shown to improve quantification accuracy [52]. Additionally, the integration of anatomical imaging modalities such as CT or MRI to assess organ volume can provide essential complementary data, allowing for more precise interpretation of functional imaging results [53].

Notwithstanding the methodological advancements that have been made, the present study is chiefly concerned with establishing an overall understanding of physiological radiotracer distribution across different developmental stages, as opposed to performing detailed partial volume effect corrections. The primary objective of our research is to assist nuclear medicine physicians in distinguishing physiological uptake patterns from pathological findings, thereby improving diagnostic accuracy. While the implementation of detailed volume-based corrections could further enhance the precision of our quantitative assessments, our findings provide a valuable reference for understanding physiological uptake trends in a developmental context. It is recommended that future studies consider incorporating more refined volume correction techniques and multimodal imaging approaches to address partial volume effect-related challenges in a more comprehensive manner. The combination of anatomical and functional imaging data will facilitate a more precise evaluation of small organ development and its effect on radiotracer distribution. This, in turn, will enhance the diagnostic and clinical applications of nuclear medicine.

A number of additional limitations of the present study merit discussion. First, at the onset of the study, a uniform dose was administered intravenously due to the absence of specific pediatric guidelines for ^68^Ga-DOTA-TATE PET/CT. Afterwards, the doses were adjusted in accordance with the updated EANM pediatric dosing card for ^68^Ga-DOTA-TATE. Nevertheless, all administered doses were administered in strict accordance with the minimum and maximum dose limits recommended by the guidelines. Second, the study was retrospective in design, and it included only subjects of one nationality, so the results may not be generalized to populations of different ethnic origins. Furthermore, the patients recruited for the study were not healthy volunteers, and some had undergone surgical resection of diagnosed tumor sites prior to imaging. Subsequently, based on clinical and imaging criteria, these patients were determined to be disease-free. Lastly, in sites where ^68^Ga-DOTA-TATE was observed for the first time, such as lymph nodes, it was not feasible to obtain tissue samples from all patients to confirm physiological normality. Instead, clinical and ancillary imaging data were utilized for this purpose.

This study demonstrates the bio-distribution pattern of ^68^Ga-DOTA-TATE based on age and location in pediatric patients. The ranges of the SUVmax and SUVmean values of ^68^Ga-DOTA-TATE obtained in the various organs are crucial for accurately diagnosing malignancy in ^68^Ga-DOTA-TATE PET/CT studies.

## Supplementary Information

Below is the link to the electronic supplementary material.

Supplementary file1 (TIF 2882 KB)

## Data Availability

The datasets generated during and/or analyzed during the current study are available from the corresponding author upon reasonable request.
