# Supplementary figures and images for: Physiological bio-distribution of 68Ga-DOTA-TATE in pediatric patients
Source: Ann Nucl Med. 2025 Mar 19;39(7):650–62. doi: 10.1007/s12149-025-02040-9 (PMC12174203; doi:10.1007/s12149-025-02040-9)

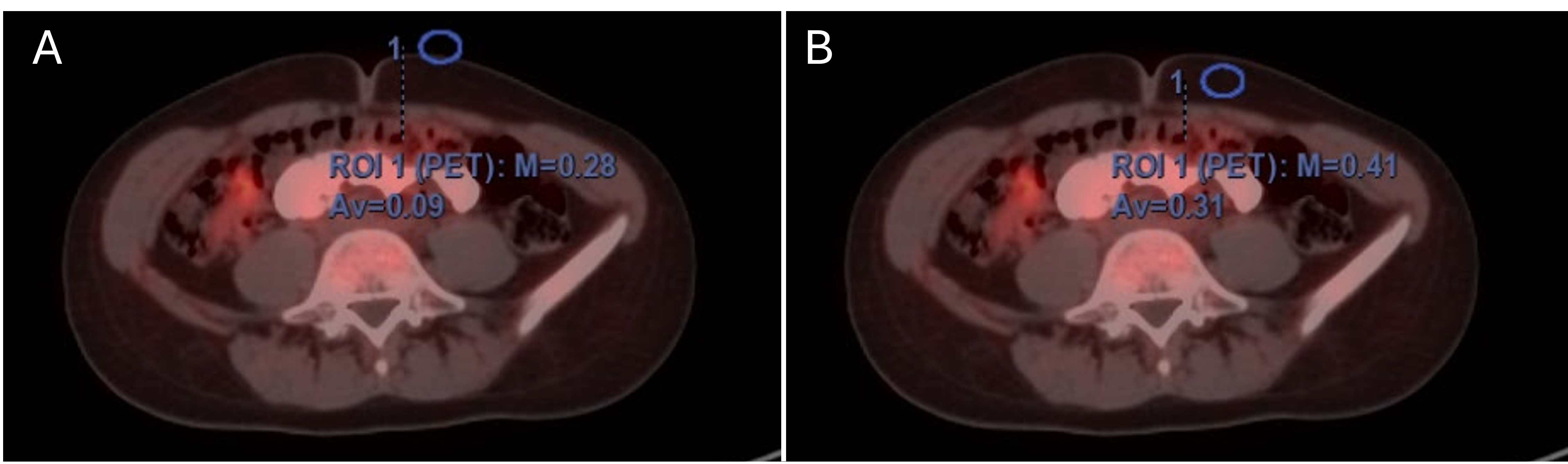

Supplement: Supplementary file 1 — Supplementary file1 (TIF 2882 KB) [file 12149_2025_2040_MOESM1_ESM.tif]
